# Body temperature measurement in ambulance: a challenge of 21-st century?

**DOI:** 10.1186/s12873-019-0261-2

**Published:** 2019-08-08

**Authors:** Paweł Podsiadło, Tomasz Darocha, Sylweriusz Kosiński, Tomasz Sanak, Robert Gałązkowski

**Affiliations:** 1 Department of Emergency Medicine, Jan Kochanowski University, IX Wieków Kielc, 19 Kielce, Poland; 2 Department of Anaesthesiology and Intensive Care, Medical University of Silesia, Medyków, 16 Katowice, Poland; 3 Faculty of Health Sciences, Jagiellonian University Medical College, Michałowskiego 12, Krakow, Poland; 4 Department of Disaster Medicine and Emergency Care, Jagiellonian University Medical College, Kopernika 19, Krakow, Poland; 5 Department of Emergency Medical Services, Medical University of Warsaw, Żwirki i Wigury 81a, Warsaw, Poland

**Keywords:** Core temperature, Thermometer, Diagnose hypothermia, Accidental hypothermia

## Abstract

**Background:**

Some crucial decisions in treatment of hypothermic patients are closely linked to core body temperature. They concern modification of resuscitation algorithms and choosing the target hospital. Under- as well as over-estimation of a patient’s temperature may limit his chances for survival. Only thermometers designed for core temperature measurement can serve as a guide in such decision making. The aim of the study was to assess whether ambulance teams are equipped properly to measure core temperature.

**Methods:**

A survey study was conducted in collaboration with the Health Ministry in April 2018. Questionnaires regarding the model, number, and year of production of thermometers were sent to each pre-hospital unit of the National Emergency Medical System in Poland.

**Results:**

A total of 1523 ground ambulances are equipped with 1582 thermometers. 53.57% are infrared-based ear thermometers, 23.02% are infrared-based surface thermometers, and 20.13% are conventional medical thermometers. Only 3.28% of devices are able to measure core body temperature. Most of analyzed thermometers (91.4%) are not allowed to operate in ambient temperature below 10 °C.

**Conclusions:**

There are only 3.28% of ground ambulances that are able to follow precisely international guidelines regarding a patient’s core body temperature. A light, reliable thermometer designed to measure core temperature in pre-hospital conditions is needed.

## Background

Human thermoregulation mechanisms lead to maintaining the gradient between the temperature of internal organs (core) and the temperature of superficial tissues (shell). Since this gradient reflects physiological thermoregulatory response, the superficial body temperature does not reflect the core temperature (Tc) [1]. A reference method for Tc measurement is the temperature of the blood in the pulmonary artery [2]. In clinical practice, the temperature measurement in the lower third of esophagus is considered to be a gold standard [3]. In a pre-hospital setting, tympanic measurement using a thermistor technique is a reliable alternative [4]. Widely available tympanic thermometers based on the infrared technique are not designed for low core temperature readings [5].

Meanwhile, some important clinical decisions in hypothermic cardiac arrest depend on core body temperature. Withholding of defibrillation after three unsuccessful attempts is recommended if Tc is below 30 °C, as well as avoiding the administration of drugs [6]. Intermittent chest compressions during evacuation in a difficult terrain are acceptable if Tc is below 28 °C. The strategy of five minutes of resuscitation alternately with five minutes of evacuation seems to be safe for severely hypothermic victims [7]. However, such pauses in moderate hypothermia may be disastrous for a victim’s brain viability. In the specific case of avalanche burial, Tc provides key information for the decision of continuing or withholding resuscitation in asystolic victims. A cut-off of 30 °C is currently used [6]. Patients in cardiac arrest due to severe hypothermia (< 28 °C) or cardiac instability and Tc < 28 °C should be transported to a hospital equipped with Extracorporeal Life Support (ECLS) [6]. The survival rate in hypothermic cardiac arrest may be as high as 100% if ECLS is applied [8]. Therefore, choosing the appropriate transport destination based on Tc can be crucial for patient survival chances.

We aimed to assess the ability of Emergency Medical Service (EMS) teams to measure core body temperature, and therefore to follow the international guidelines pertaining to the treatment of hypothermic victims.

## Methods

This cross sectional study was conducted in collaboration with the Health Ministry in April 2018. The questionnaire was sent to all 180 operators of the EMS system in Poland. The form was to be filled out with the number and the models of thermometers, as well as the year of production and the manufacturer’s name. Thermometers were divided into two main groups, namely those suitable for measuring core body temperature (Tc thermometers), and others (non-Tc thermometers). Only devices based on the thermocouple or thermistor, and dedicated to esophageal, rectal, bladder or epitympanic measurement were classified as Tc thermometers. All other devices were analysed in three subgroups regarding their construction: conventional clinical thermometers designed to measure surface temperature (for example, in the axilla or in the mouth), infrared-based ear thermometers, and infrared-based contactless skin thermometers. The technical data of all reported models was completed. Since pre-hospital care is often provided in cold environment, the operating temperature of every device was investigated in the user’s manual, as well as the lower limit of the measured temperature range. The median length of service of the analysed thermometers was calculated based on the year of production.

The data are presented based on descriptive statistics, namely numbers and percentages.

The consent of the Ethical Board was not required as no patient medical records were used.

## Results

There are 1523 ground ambulances in Poland governed by 180 operators (official data of Ministry of Health). All operators returned completed questionnaires with a total of 1582 thermometers (96 models) being reported. The data of 57 devices (3.6%) were deemed incomplete and excluded from detailed analysis (all these devices were infrared-based thermometers). The number of thermometers in particular categories and their measured temperature ranges are showed in Table 1.

**Table 1:** Construction, number, and measurement range of analysed thermometers

	Classical clinical thermometers			Skin (infrared) thermometers			Ear (infrared) thermometers			Thermometers designed to Tc measurement			Total
										Cardiomonitor-dedicated probe		Autonomous devices	
Lower limit of measurement range (°C)	< 28	≥28	≥32	< 28	≥28	≥32	< 28	≥28	≥32	Model A	Model B	17	
										24,8	0		
*n*	9	135	163	158		193	711		106	27		23	1525
Total in category	307 (20.13%)			351 (23.02%)			817 (53.57%)			50 (3.28%)			

The majority of thermometers (91.4%) are not designed to operate in an ambient temperature below 10 °C (Table 2).

**Table 2:** Operating temperature of analysed thermometers

Lower limit of operating temperature	Number of thermometers	
	Non-Tc thermometers	Tc thermometers
< 0 °C		23 (46%)
0 °C	3 (0.20%)	27 (54%)
5 °C	78 (5.29%)	
10 °C	1103 (74.78%)	
≥15 °C	291 (19.73%)	
Total	1475 (100%)	50 (100%)

The median length of service of the analysed thermometers is 3 years, (IQR [2, 4]).

The distribution of Tc thermometers in particular regions of the country reflects neither the climatic conditions nor the local infrastructure.

## Discussion

Our study showed that only 3.28% of Polish ground ambulances are equipped adequately to measure core body temperature.

Precise assessment of core temperature is necessary for making some vital decisions in the treatment of severe hypothermia. Unfortunately, more than half of all thermometers in Polish ambulances are infrared-based ear devices. Although epitympanic measurement appears to be an appropriate method of Tc assessment in hospital settings, the impact of environmental factors, such as cold and wind, substantially affects its accuracy. The epitympanic method is also unreliable in cardiac arrest due to lack of blood flow in the tympanic artery. Only thermistor-based devices with an insulating seal, when the ear canal is unobstructed and dry, may provide a reliable Tc reading [9, 10].

Although some conventional, thermistor-based thermometers with a stiff metallic probe could be used for rectal measurement, perforation of the rectum is a major concern [11]. The actual core temperature can be taken with the use of flexible rectal probes that should be inserted to a depth of circa 15 cm. Such a measurement is technically difficult and associated with undressing the patient which is not advisable in pre-hospital cold environment. Autonomous Tc thermometers reported in this study are adjusted to a thin and excessively flexible probe that placement seems not convenient.

The study regarding the equipment of Swedish rescue services showed that 22% of ground ambulances have Tc thermometers, including 13% of ear thermistor-based devices and 9% for rectal use [12]. In Norway, although 12% of ground ambulances are equipped with hypothermia thermometers, most of them are designed for rectal use [13].

The operating temperature range seems to be an important limitation of the use of thermometers in pre-hospital settings. The mean annual temperature in Poland ranges from 6 °C to 9 °C while the mean winter temperature ranges from − 5 °C to 0 °C depending on the region (data of Polish Meteorological Institute). The majority of analysed thermometers are allowed to operate in an ambient temperature ≥ 10 °C according to their users’ manuals. This makes them unsuitable for outdoor use, especially in cold weather conditions, when patients are likely to suffer from accidental hypothermia.

In 2003 Durrer et al. proposed a Swiss Staging System to estimate Tc when suitable thermometers are lacking [14]. This classification is based on clinical symptoms and easy to apply. However, many patients can be misclassified due to individual variability and a lack of correspondence between clinical signs and core temperature which occurs in about 50% of cases [15]. This may affect the decision-making process regarding target hospitals for patients in cardiac arrest, and delay their arrival to an ECLS facility. A consequence of overestimating Tc may be the referral of a severely hypothermic patient to the nearest hospital where ECLS is not available, thereby decreasing their chances for survival. This is very likely when a patient with Tc below 28 °C presents vital signs mimicking moderate hypothermia [16].

Accidental hypothermia seems to be a rare problem – it is a cause of 1500 deaths per year in the United States [17]. However, this is the only reversible cause of cardiac arrest that allows up to 100% of patients to survive without neurological deficit [8].

Future research would be welcome to develop a medical thermometer that would be validated at low ambient temperatures, low Tc, as well as being also light, strong and inexpensive.

Since the findings of our study are based on the data from a single country, their generalizability is limited. However, results of analogous studies in Norway, Sweden, and United Kingdom are similar.

## Conclusions

Most ground ambulances are not able to measure patient’s core temperature. Thus, it is impossible to follow the resuscitation guidelines without the risk of worsening the outcome.

It would be beneficial to equip rescue services with a reliable low-reading thermometer which would be cheap to use and designed to be operated in winter conditions.

## Data Availability

The datasets used and/or analyzed during the current study are available from the corresponding author on reasonable request.

## Abbreviations

ECLS: Extracorporeal Life Support
EMS: Emergency Medical Service
Tc: Core temperature
